# Congenital and childhood atrioventricular blocks: pathophysiology and contemporary management

**DOI:** 10.1007/s00431-016-2748-0

**Published:** 2016-06-28

**Authors:** Alban-Elouen Baruteau, Robert H. Pass, Jean-Benoit Thambo, Albin Behaghel, Solène Le Pennec, Elodie Perdreau, Nicolas Combes, Leonardo Liberman, Christopher J. McLeod

**Affiliations:** 1 Cardiovascular and Cell Sciences Research Center, St George’s University of London, London, UK; 2 LIRYC Institute, CHU Bordeaux, Department of Pediatric Cardiology, Bordeaux-II University, Bordeaux, France; 3 Service de Cardiologie Pédiatrique, Hôpital du Haut Lévèque, Institut Hospitalo-Universitaire LIRYC (Electrophysiology and Heart Modeling Institute), 5 avenue de Magellan, 33600 Pessac, France; 4 Division of Pediatric Electrophysiology, Albert Einstein College of Medicine, Montefiore Children’s Hospital, Bronx, NY USA; 5 CHU Rennes, Department of Cardiology, LTSI, INSERM 1099, Rennes-1 University, Rennes, France; 6 Department of Cardiology, Clinique Pasteur, Toulouse, France; 7 Morgan Stanley Children’s Hospital, Division of Pediatric Cardiology, New York Presbyterian Hospital, Columbia University Medical Center, New York, NY USA; 8 Mayo Clinic, Division of Cardiovascular Diseases, Mayo Clinic College of Medicine, Rochester, MN USA

**Keywords:** Heart block, Pacemaker, Pathophysiology, Outcomes, Congenital heart disease

## Abstract

Atrioventricular block is classified as congenital if diagnosed in utero, at birth, or within the first month of life. The pathophysiological process is believed to be due to immune-mediated injury of the conduction system, which occurs as a result of transplacental passage of maternal anti-SSA/Ro-SSB/La antibodies. Childhood atrioventricular block is therefore diagnosed between the first month and the 18th year of life. Genetic variants in multiple genes have been described to date in the pathogenesis of inherited progressive cardiac conduction disorders. Indications and techniques of cardiac pacing have also evolved to allow safe permanent cardiac pacing in almost all patients, including those with structural heart abnormalities.

*Conclusion*: Early diagnosis and appropriate management are critical in many cases in order to prevent sudden death, and this review critically assesses our current understanding of the pathogenetic mechanisms, clinical course, and optimal management of congenital and childhood AV block.

**Table:** 

**What is Known:** *• Prevalence of congenital heart block of 1 per 15,000 to 20,000 live births. AV block is defined as congenital if diagnosed in utero, at birth, or within the first month of life, whereas childhood AV block is diagnosed between the first month and the 18th year of life. As a result of several different etiologies, congenital and childhood atrioventricular block may occur in an entirely structurally normal heart or in association with concomitant congenital heart disease. Cardiac pacing is indicated in symptomatic patients and has several prophylactic indications in asymptomatic patients to prevent sudden death.* *• Autoimmune, congenital AV block is associated with a high neonatal mortality rate and development of dilated cardiomyopathy in 5 to 30 % cases.*
**What is New:** *• Several genes including SCN5A have been implicated in autosomal dominant forms of familial progressive cardiac conduction disorders.* *• Leadless pacemaker technology and gene therapy for biological pacing are promising research fields. In utero percutaneous pacing appears to be at high risk and needs further development before it can be adopted into routine clinical practice. Cardiac resynchronization therapy is of proven value in case of pacing-induced cardiomyopathy.*

## Background

Cardiac conduction disorders are rare syndromes in neonates and children [15, 64]. As a result of several different etiologies, it may occur in an entirely structurally normal heart or in association with concomitant congenital heart disease (CHD). In contrast to acquired atrioventricular (AV) conduction block, congenital heart block (CHB)—identified in utero in normal hearts—holds a significantly different prognosis with an increased risk of late-onset cardiomyopathy. Fundamentally, the pathogenesis is also disparate, driven by different maternal clinical features and an increased risk of recurrence in future pregnancies. For these reasons, AV block is classified as congenital if diagnosed in utero, at birth, or within the first month of life. Therefore, childhood AV block is diagnosed between the first month and the 18th year of life [15]. The estimated prevalence of congenital heart block is 1 per 15,000–20,000 live births [64].

## Atrioventricular conduction disorders in structurally normal hearts

### Immune-mediated AV block

Although some aspects remain to be clarified, pathophysiology, therapeutic approach, and long-term prognosis of immune-mediated AV block have been extensively studied, as it is one of the leading causes of congenital heart blocks.

#### Pathophysiology

Congenital AV block can be passively acquired via an autoimmune process affecting the developing heart due to the transplacental passage of maternal anti-Ro/SSA and/or anti-La/SSB autoantibodies. Entering the fetal circulation, they can directly bind L-type calcium channels on fetal cardiomyocytes and significantly, but reversibly, inhibit the related currents. However, in some cases, for unclear reasons, the prolonged exposure to anti-Ro/SSA antibodies may induce calcium channel internalization, in turn triggering a complex and only in part mechanistically known perturbation of the cytoplasmic calcium metabolism which ultimately leads to apoptosis and cell death, and then to local inflammation. If the process is not stopped in this phase, the inflammatory damage proceeds, thus eventually resulting in fibrosis and calcification of the cardiac conduction system. This mechanistic sequence, also known as “calcium channel hypothesis,” is currently recognized as the more attractive theory possible explaining the pathogenesis of the disease [1, 90]. Maternal autoantibodies can be detected in over 95 % of fetuses or newborns presenting with AV block, namely congenital AV block [19]. In contrast, maternal autoantibodies have been detected in only a minority of children, in whom AV block was diagnosed beyond the neonatal period, a different, distinct clinical entity [15, 34, 95]. However, some isolated AV blocks diagnosed beyond the neonatal period are also immune-mediated, even with late detection of maternal anti-Ro/SSA autoantibodies [12]. This condition, emerging in childhood or even in the adult age, represents a late progressive congenital form of immune AV block with the late development of a subclinical anti-Ro/SSA-induced congenital damage of the conduction system, related to a not fully understood autoantibody-independent worsening with age [51]. Between 2 and 5 % of fetuses and infants whose mothers are autoantibody-positive develop AV block, and the risk to subsequent pregnancies is substantial (ranging between 12 and 25 %) in mothers who have had a child with congenital AV block [16]. In up to a third of infants with congenital AV block, a characterized autoimmune disease, such as lupus, is present in the mother [16]. However, in the large majority of the cases, AV block occurs in fetuses of healthy, silently anti-Ro/SSA antibodies-carrying mothers. The diagnosis of mothers’ seropositivity is thus usually made only after congenital AV block detection. In a recent prospective study of 186 antibody-exposed fetuses and infants, it has been demonstrated that mothers of children with cardiac involvement were less likely to have had a connective tissue disease than mothers of children without cardiac involvement [36].

#### Diagnosis of fetal AV block

Fetal echocardiography is the gold standard for the diagnosis of congenital AV block. All M-mode and Doppler echocardiographic techniques rely on the relationship between atrial and ventricular mechanical event [30]. Although clinical applications of both fetal electrocardiography and fetal magnetocardiography are more recent, these two noninvasive tools are promising, being able to more precisely diagnose fetal arrhythmias and conduction disorders [23, 103]. Complete fetal AV block develops during gestational weeks 16 to 24, although a later onset of the phenomenon up to gestational week 34 has been described [2, 72].

#### Clinical course

CHB derived from an autoimmune process is associated with a high neonatal mortality rate [16, 37]. The estimated overall mortality without pacing is estimated to be around 8–16 % in infants and half as much in children and adults [37, 65]. Interestingly, cardiac dilatation and impaired ventricular function can develop as long-term sequelae in those who forego pacing and those who are permanently paced. Without pacing support, it appears that the slow heart rates and associated higher stroke volumes probably drive this process [65]. But with pacing, the current hypothesis centers on a pacing-induced cardiomyopathy. Globally, the prevalence of dilated cardiomyopathy (DCM) in CHB ranges from 5 to 30 % [66, 91, 94]. Various pathophysiological processes, including transient myocarditis or immune-mediated myocardial injury, have been proposed to explain the development of ventricular dilatation and dysfunction. Late-onset dilated cardiomyopathy in patients with complete heart block may be a sequela of in utero autoimmune myocarditis or due to its postnatal reactivation [95]. Right and left ventricular endocardial fibroelastosis and fibrosis have been observed at autopsy. These observations were not limited to the conduction system and involved the working ventricular myocardium. The detrimental effects of maternal antibodies directed against fetal cardiac tissue provided evidence in favor of the immunopathologic role played by the maternal autoantibodies in congenital AV block. In view of the myocardial dystrophic changes and adverse remodeling caused by ventricular desynchronization, right ventricular pacing has been suggested as an important cause of DCM [66, 91, 94]. In some patients, the discontinuation of right ventricular pacing or upgrade to cardiac resynchronization therapy alone normalized systolic function, which would not be expected to occur if ongoing myocarditis or other autoimmune factors were the only cause of DCM [13, 39, 67].

### Inherited AV block

Inherited progressive cardiac conduction disease (PCCD) is diagnosed in patients less than 50 years of age with an unexplained progressive conduction abnormality but with an otherwise structurally normal heart, especially if there is a family history of PCCD. This excludes the skeletal myopathies and muscular dystrophies, given the recognized impact of such progressive disorders on the cardiac muscle [70]. Since the publication of Morquio’s first report of familial segregation of heart blocks in 1901, major advances have been made in our understanding of the clinical, genetic, and molecular characteristics of inherited PCCD. Familial clustering of PCCD of unknown cause, including congenital AV block, has been reported. Published pedigrees have shown an autosomal dominant inheritance with incomplete penetrance and variable expressivity [32, 56]. Inherited PCCD in structurally normal hearts presents as a primary electrical disease and has been linked to genetic variants in the ion channel genes *SCN5A*, *SCN1B*, *SCN10A*, *TRPM4*, and *KCNK17* as well as in genes coding for cardiac connexin proteins [8, 58, 77]. Moreover, *SCN5A* mutation carriers tend to exhibit “cardiac sodium channelopathy overlap syndrome,” with overlapping clinical manifestations of the distinct *SCN5A*-related syndromes such as long QT syndrome type 3 or Brugada syndrome, and an altered cardiac conduction in many cases [44]. It is now clear that complex pathophysiological processes involving many genes and gene networks may lead to the occurrence of atrioventricular and intraventricular block. It is likely that only a small fraction of these genetic defects have been identified, and it is likely that genetic tests could help in the future to better determine the risk of progression of a conduction defect and hence determine the best timing for pacemaker implantation.

### Apparently “idiopathic” AV block

Rarely, AV block of unknown origin appears during childhood, in the absence of maternal antibodies, structural heart disease, or other overt causes. Scientific literature is scarce regarding the etiology and the clinical course of these patients with apparently idiopathic heart block. In the first large-scale study, looking for heritability of pediatric idiopathic heart block in a French nationwide cohort, Baruteau et al. observed a high degree of inheritance and a strong genetic background in the pathogenesis of congenital and childhood nonimmune isolated AV block [9, 10]. Thus, familial screening should be considered and may provide strong arguments for heritability, even in patients where the disorder appears to be sporadic and idiopathic.

## Atrioventricular conduction disorders in association with congenital heart disease

### Native

Recent genetic findings suggest that approximately 10 % of sporadic CHD may have de novo mutations that significantly contribute to the disease process [101]. Mutations in genes encoding for transcription factors critical for cardiac chamber formation, endocardial cushion remodeling, and conduction system development, like *NKX2.5* and *Tbx5*, may lead to PCCD associated with CHD [61]. Numerous mutations in *NKX2.5* have been reported with various CHD phenotypes, such as secundum atrial septal defect, tetralogy of Fallot, truncus arteriosus, double-outlet right ventricle, L-transposition of great arteries, interrupted aortic arch, ventricular noncompaction, and hypoplastic left heart, with or without conduction disorders [62, 78]. *Tbx5* mutations are responsible for Holt-Oram syndrome, an autosomal dominant inherited disease characterized by radial ray upper limb abnormalities, cardiac septation defects, and various degrees of cardiac conduction disorders which may occur even in the absence of overt structural heart disease [6].

Kearns-Sayre syndrome is a mitochondrial disorder characterized by onset before the age of 20, progressive external ophthalmoplegia, and pigmentary retinopathy, accompanied by either cardiac conduction defects, elevated cerebrospinal fluid protein, or cerebellar ataxia. Fifty percent of affected patients develop cardiac complications, the most common of them being conduction disease which may progress to complete AV block or bradycardia-related polymorphic ventricular tachycardia [42].

Heart block affects one third of fetuses with heterotaxy syndrome and left atrial isomerism, being a primary risk factor for perinatal mortality [88]. The most common CHD associated with conduction disorders is L-transposition of the great arteries [97]. Abnormal development of the central fibrous body with lack of union between AV node and AV bundle or formation of the conduction tissue from the anterior endocardium were suggested to be the possible causes of block seen in L-transposition [3]. The lifelong risk for complete block in these patients is roughly 1 % annually and roughly 50 % to develop heart block spontaneously by age 50 [97].

### Postoperative

**Fig. 1:**
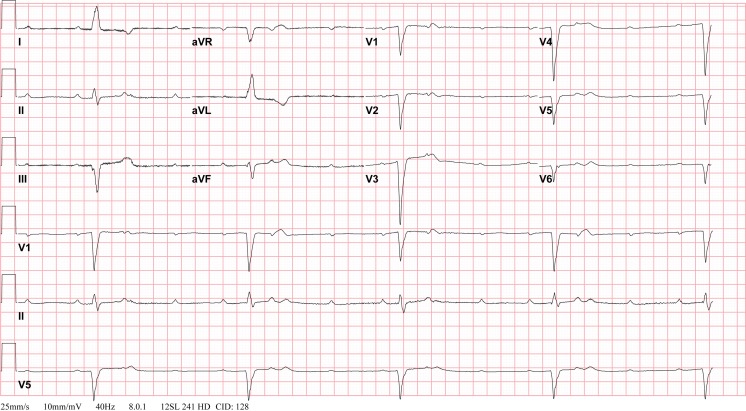
Complete atrioventricular block: unpaced electrocardiogram. Postoperative 12-lead electrocardiogram demonstrating complete heart block with slow ventricular escape rate, after tricuspid valve replacement

**Fig. 2:**
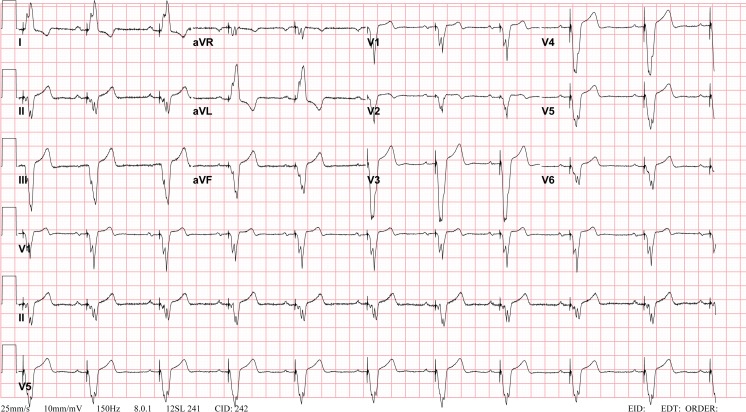
Complete atrioventricular block: paced electrocardiogram. Twelve-lead electrocardiogram from patient demonstrating atrial sensed ventricular paced rhythm

Following CHD surgery, any degree of AV block may be seen (Figs. 1 and 2). A retrospective multicenter study recently evaluated incidence of postoperative complete heart block in children undergoing congenital heart surgery [53]. Among 103,616 surgeries from 45 US tertiary care hospitals, the incidence of complete heart block requiring pacemaker placement was low (1.2 %), mainly associated with mitral valve repair or replacement (3.7 %), aortic valve repair or replacement (2.7 %), atrioventricular canal surgery (1.9 %), and ventricular septal defect (VSD) surgery (1.8 %). However, these patients incurred longer hospital stay and had higher mortality even after accounting for heart surgery complexity.

In roughly one third of the cases of postoperative complete heart block, AV conduction does not recover and those patients should undergo pacemaker implantation. Permanent pacemaker implantation should be considered in all patients who have postoperative high-grade AV block following CHD surgery that exceeds 7–10 days, even in the setting of a narrow QRS escape rate [14, 26]. During this period, temporary pacing wires may be necessary to maintain adequate chronotropy. Postoperative heart block has also been rarely reported in patients who had been previously discharged from the hospital with normal AV conduction after open-heart surgery. Close and continued follow-up of postoperative CHD surgical cases, particularly VSD, is necessary due to the risk of possible progression of block over time [54].

## Atrioventricular conduction disorders in association with acquired heart disease

AV block in the young can also be derived from a wide variety of causes such as surgical or catheterization-induced trauma, coronary artery disease, acute or chronic infectious processes, myocarditis, hypersensitivity cardiomyopathy, metabolic abnormalities, hypothyroidism, infiltrative processes, or through a pathological neurocardiogenic mechanism [7]. Even if temporary pacing might be required in unstable patients with Lyme carditis, complete heart block is usually reversible with appropriate antibiotics [28]. Chagas disease is an endemic disease in most Latin American countries, and around one third of affected patients develop cardiac conduction disorders requiring pacemaker implantation [4]. Incidence of catheterization-induced heart block was recently evaluated at 2.2 %, with a high rate of recovery following a similar course to that of postsurgical heart block [57]. Some interventional procedures, such as device closure of perimembranous VSD and catheter ablation of AV nodal reentrant tachycardia or parahissian accessory pathways, carry a risk of permanent heart block [49, 100, 102]. The incidence of AV node dysfunction is apparently higher in patients with Kawasaki disease, possibly caused by myocarditis or an abnormal microcirculation in the AV node artery. Acute rheumatic carditis must also be kept in mind in the diagnostic work-up of patients with AV conduction disorder in association with acquired heart disease, particularly when it occurs in pediatric patients; Most cases are reversible first- or second-degree AV blocks. Although a rare and usually transient finding during acute rheumatic fever, complete AV block may lead to symptoms and need a specific treatment.

## Management

### Treatment options during fetal life

Left untreated, congenital AV block is associated with a fetal and neonatal mortality ranging between 14 and 34 % [76]. Fetal hydrops and ventricular escape rates <55 bpm have been identified as risk factors for mortality [54, 55]. Transplacental treatment options are not consensual. Dexamethasone use may significantly lower fetal mortality [38], but its administration remains controversial because of its potential side effects for both mother and fetus, especially potential fetal neurological development impairment [68]. Maternal administration of terbutaline has also been reported, used alone or in addition to dexamethasone [20]. Although largely controversial, prenatal treatment may also include intravenous immunoglobulins and plasmapheresis, used alone or together in combination with steroids [31, 75].

Using the currently available techniques, in utero percutaneous pacing appears to be at high risk, with fetal deaths occurring within a few hours of the procedure in a high proportion of cases [5]. Further studies would be required to improve our understanding of the natural history of congenital AV block in order to identify more accurately the fetuses at highest risk. Development in the techniques and technologies available to deliver in utero pacing would also be required before this treatment can be adopted into routine clinical practice.

### Postnatal medical therapy

Pharmacological therapy has a distinct role in the acute management of severe bradycardia (whether sinus- or AV nodal-related) and should typically be carried out alongside parallel efforts to arrange for transcutaneous pacing and temporary cardiac pacing in order to prevent from sudden cardiac death [48]. Intravenous isoproterenol, atropine, epinephrine, and dopamine are all recommended [48]. Beyond the acute management in this context, no medication is proven to improve chronic sinus- and AV nodal function. It is crucial, however, to recognize the potential for medications to compromise cardiac conduction, especially considering the atypical antihypertensive and antipsychotic agents, in addition to the AV nodal blocking agents they may contain. Monitoring of children who do not require neonatal pacing is based on 24-h Holter ECG and transthoracic echocardiography that should be performed frequently.

### Permanent pacemaker implantation

The indications for permanent pacing in children or adults with CHD are similar to those recommended in acquired heart disease, yet there are important differences in the approach based primarily on anatomy and somatic growth.

#### Indications

In essence, every symptomatic, non-reversible AV node disease requires permanent pacemaker implantation [14, 26]. Pacing must also be considered in asymptomatic high degree AV blocks with specific risk conditions. Recent indications for cardiac pacing in children and CHD patients are summarized in Table 1. Although historical series of isolated congenital AV block reported a high incidence of unpredictable Stokes-Adams attacks and a high mortality associated with the first attack [2, 18], latest studies performed in our era of pediatric cardiac pacing showed that prophylactic pacing, as currently recommended [44, 45], is associated with strong reduction in the morbidity and mortality due to Stokes-Adams attacks [32].

**Table 1:** Pacing indications in children and patients with congenital heart disease

	ESC guidelines	ACCF/AHA/HRS guidelines
Congenital AV block		
Symptomatic advanced second- or third-degree AV block	Class I, level C	Class I, level C
Asymptomatic high degree AV block with ventricular dysfunction	Class I, level C	Class I, level B
Asymptomatic high degree AV block with prolonged QTc interval	Class I, level C	–
Asymptomatic high degree AV block with complex ventricular ectopy	Class I, level C	Class I, level B
Asymptomatic high degree AV block with wide QRS escape rhythm	Class I, level C	Class I, level B
Asymptomatic high degree AV block with abrupt ventricular pauses >threefold the basic cycle length	Class I, level C	Class IIa, level B
Asymptomatic third-degree AV block in the infant with a ventricular rate <55 bpm or with CHD and a ventricular rate <70 bpm	–	Class I, level C
Third-degree AV block beyond the first year of life with an average heart rate <50 bpm	–	Class IIa, level B
Asymptomatic high degree AV block with a ventricular rate <50 bpm	Class I, level C	–
Third-degree AV block beyond the first year of life with symptoms due to chronotropic incompetence	–	Class IIa, level B
High degree AV block in asymptomatic children/adolescents in absence of the above risk conditions	Class IIb, level C	Class IIb, level B
Asymptomatic type I second-degree AV block	–	Class III, level C
Postoperative AV block		
Postoperative advanced second- or third-degree AV block that persists >7 days after cardiac surgery (10 days in ESC guidelines)	Class I, level B	Class I, level B
Transient postoperative third-degree AV block that reverts to sinus rhythm with residual bifascicular block	Class IIa, level C	Class IIb, level C
Unexplained syncope in the patient with prior CHD surgery complicated by transient complete heart block with residual fascicular block	–	Class IIa, level B
Transient postoperative AV block with return of normal AV conduction in the otherwise asymptomatic patient	–	Class III, level B
Asymptomatic postoperative bifascicular block with/without first-degree AV block in the absence of prior transient complete AV block	–	Class III, level C

Levels of evidence are classified in “level A” if data are derived from multiple randomized clinical trials or meta-analyses, “level B” if data are derived from a single randomized clinical trial or large non-randomized studies, and “level C” if there is a consensus of opinion of the experts and/or if data are derived from small studies, retrospective studies, or registries. Recommendations are listed according to the commonly used class I, IIa, IIb, and III classification and the corresponding language: “is recommended” for a class I recommendation; “can be useful” for a class IIa recommendation; “may be considered” to signify a class IIb recommendation; and “should not” or “is not recommended” for a class III recommendation. ESC guidelines: reference [44]; ACCF/AHA/HRS guidelines: reference [45]

*AV* atrioventricular, *CHD* congenital heart disease

#### Endocardial versus epicardial device

**Fig. 3:**
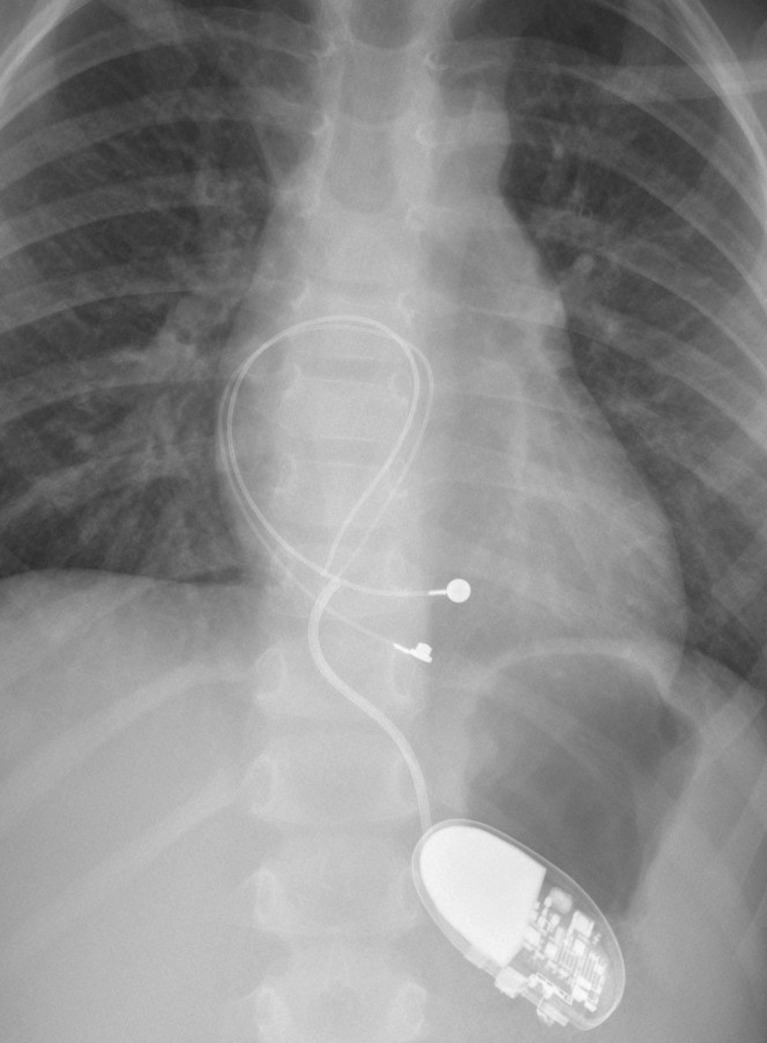
Permanent pacemaker with epicardial leads (VVI pacing)

**Fig. 4:**
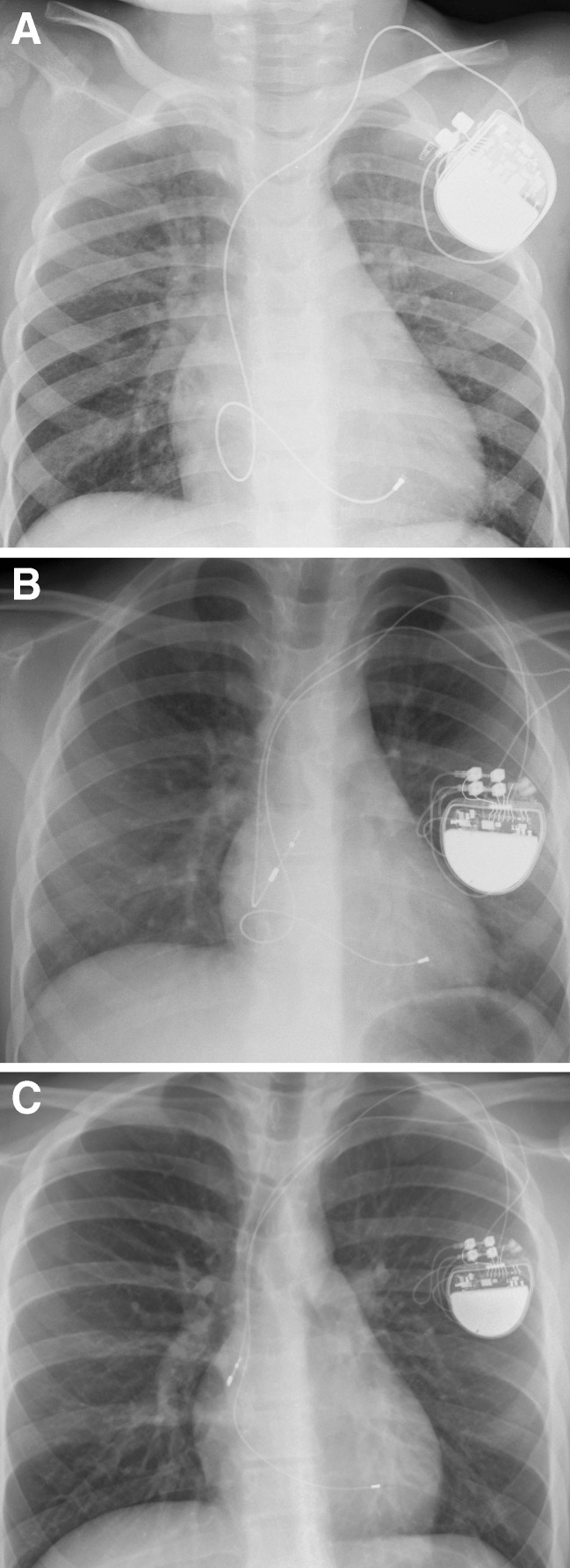
Permanent pacemaker with transvenous leads. Growth and change in a loop of an endocardial lead. A 4-year-old boy with childhood isolated nonimmune atrioventricular block underwent pacemaker implantation using a transvenous lead. Radiographs at 4 years of age (**a**, VVI pacing), 6 years later (**b**, DDD pacing), and 9 years later (**c**, DDD pacing). Note the change in the loop of the lead as the child grows

**Fig. 5:**
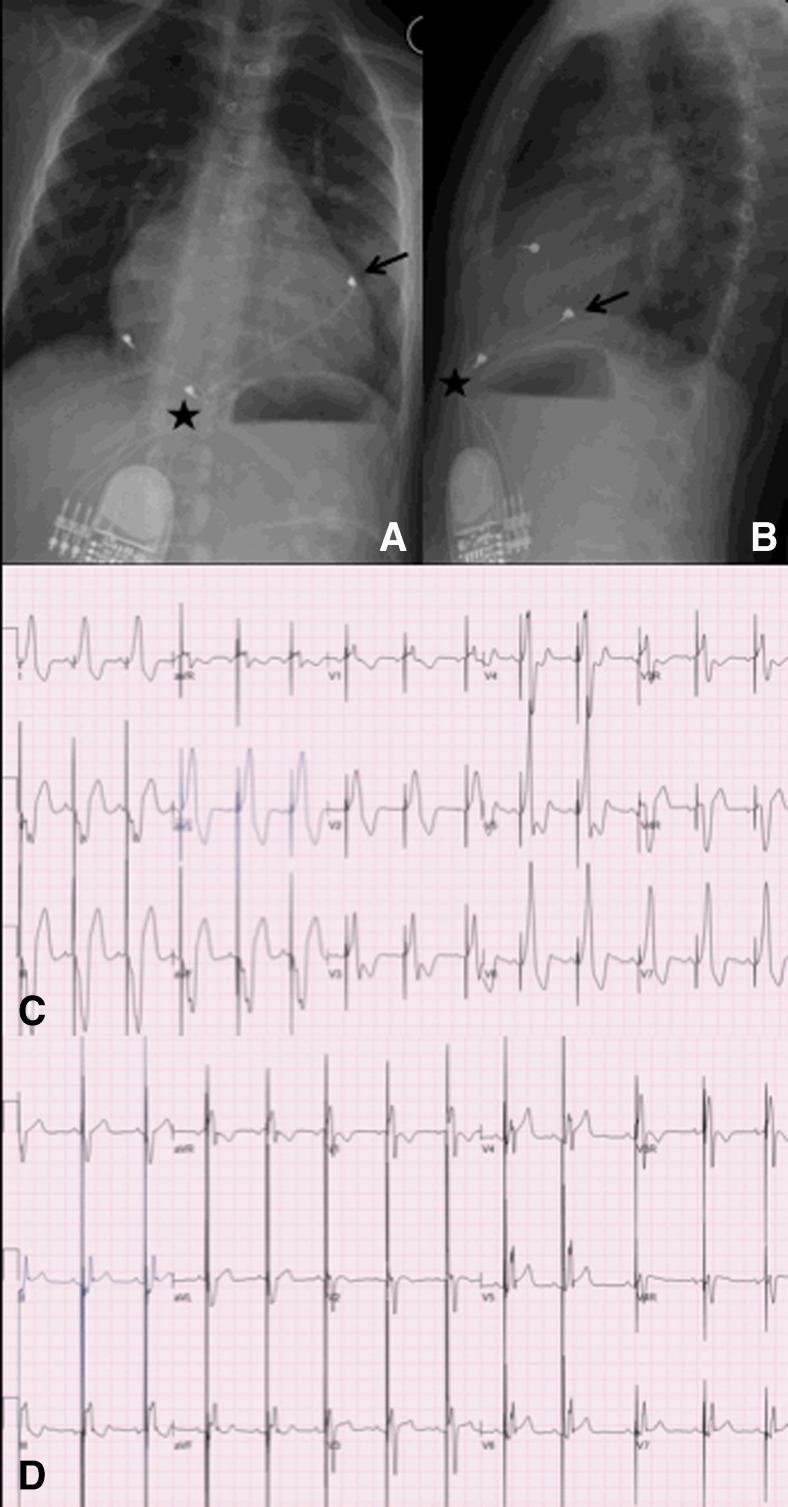
Cardiac resynchronization therapy with epicardial leads. A 5-year-old boy who underwent a neonatal Ross procedure had postoperative complete heart block and left ventricular dysfunction. He was implanted with epicardial multisite pacing with right atrial, right ventricular (**a** and **b**, *black star*), and left ventricular (**a** and **b**, *black arrow*) leads. Biventricular pacing allow shortening of the paced QRS (**c**, 224 ms) compared with right ventricular pacing alone (**d**, 128 ms)

**Fig. 6:**
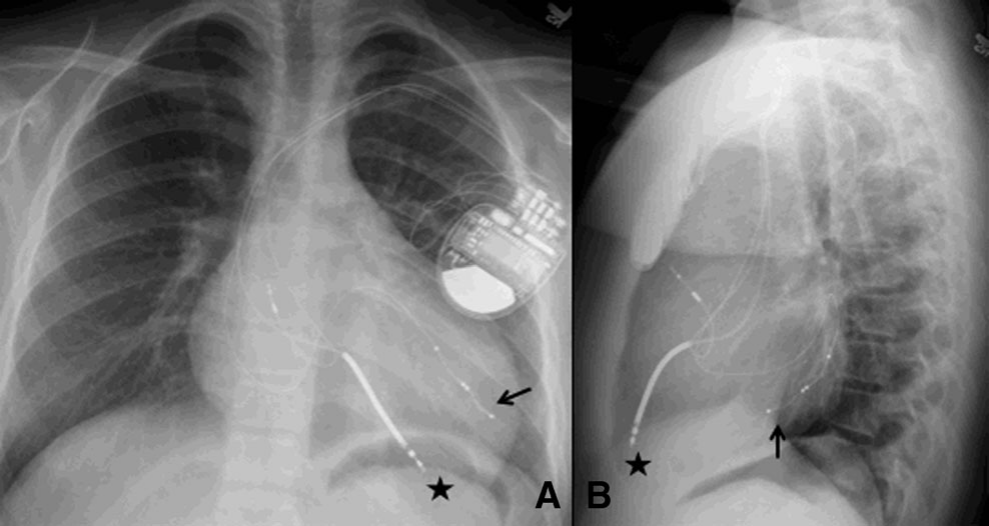
Cardiac resynchronization therapy with transvenous leads. A 14-year-old boy with dilated cardiomyopathy and left ventricular dysfunction, second-degree AV block, and an episode of ventricular fibrillation had implantation of a biventricular implantable cardioverter-defibrillator with a transvenous right ventricular lead (**a** and **b**, *black star*) and a transvenous left ventricular lead into the coronary sinus (**a** and **b**, *black arrow*)

Although pacing indications are clearly defined, whether an endocardial versus an epicardial system is preferred remains to be clarified [26, 48, 96]. The large population of adult paced patients allows for surgical practices to be studied more effectively and lead to evidence-based transitions in patient care. Meanwhile, the smaller volume of paced pediatric patients does not provide enough information and clinical decisions based on faith, opinion, experience, and some retrospective data. Within those limitations, there is a general consensus that the smallest infants are best served with epicardial pacing systems, with a cutoff weight around 15–20 kg [26, 98]. Epicardial leads are more likely to fracture and are prone to exit block, and implantation requires a major operation that is accompanied by the inherent risks and need for perioperative support [89] (Fig. 3). However, many groups standardly use epicardial pacing in infants and young children and argue that endocardial systems may carry a significant risk of venous thrombosis in infants, which can result in loss of venous access in the future, leading to a more complicated lead revision later in the patient’s life [18, 29, 50]. Up to a 19 % transvenous lead-related failure rate has been reported by others who choose the endocardial approach [80]. Extraction of abandoned transvenous leads in the pediatric population is also problematic, and optimal lead management still remains to be defined [17, 60]. On the other hand, there is a global trend towards using endocardial leads in younger patients and some institutions actively implant transvenous leads in children weighing less than 15 kg [43, 63, 73, 79, 83, 85]. It has been shown that an 80-mm right atrial lead loop will allow 6 to 12 years (mean, 8 years) of growth in infants and children without the need for reoperation to adjust lead length [33] (Figs. 4, 5 and 6). Long-term follow-up demonstrates that the longevity of an endocardial system exceeds that of its epicardial counterpart [89, 98], also an important consideration in patients who will be exposed to the cumulative burden of repeated lead and device reimplantation. Despite growing experience, endocardial implantation is not universally accepted. Most publications reporting results of transvenous pacing in infants and young children are from small and/or older studies [33, 40, 43, 55], so that long-term follow-up data from large patient populations should still be clarified.

#### Route of pacing

Gaining access to the chamber requiring pacing is another central hurdle, frequently challenging in this patient group, and thereby commonly dictating the route of pacing but also highlighting why a detailed surgical history is vital. Modern-day pacemaker implantation is therefore suitably complemented by adjunctive CT or MR angiography, indicating whether an endocardial system is possible, and also providing a map for coronary sinus lead placement. For complex CHD patients with prior operative intervention and whose surgical reports are not available, venography at the time of the procedure is recommended [14, 26]. Absolute contraindications to conventional transvenous, endocardial lead placement include occlusion of the superior vena cava (SVC) (bidirectional cavopulmonary anastomosis/Glenn), extra-cardiac Fontan procedures, baffle thrombosis, and severe baffle stenosis. Attempts to access the subpulmonic ventricle by crossing a mechanical atrioventricular valve should also absolutely be avoided [27]. In this situation, coronary sinus lead placement can potentially be used for ventricular pacing.

In patients in whom a conventional transvenous approach via the SVC is not possible, femoral and transhepatic lead implantation can also be considered. Femoral techniques have been reported predominantly in children, suggesting this is a viable alternative with issues related primarily to the stability of the atrial lead and discomfort from the abdominally placed generator [59]. This approach does entail a higher incidence of lead failure given the additional mechanical stress associated with hip flexion. Transhepatic implants have also only been reported in children and acutely present the additional risks of intraabdominal or intracapsular hemorrhage, and long-term outcomes remain uncertain [25].

Placing leads in patients with prior atrial switch operations (Mustard/Senning) must be handled with utmost care, given the frequency of baffle leaks in this group [47]. Pre-emptive covered stent implantation or even open repair should be considered given the risk of thromboembolism across these veno-systemic shunts. A transcatheter approach is utilized for leak closure but can also be applied for dilatation of a narrowed baffle that would otherwise be occluded by transvenous lead implantation [11, 24].

#### Thromboembolism

Any patient with an intracardiac shunt is at higher risk for thrombus formation on transvenous pacing leads and subsequent systemic thromboembolic complications [21, 45]. Pre-emptive transesophageal echocardiography with bubble injections is therefore critical, and if any shunt is seen, the approach should be modified. If the shunt can be closed, either via an open approach or using a percutaneous approach, then this should be undertaken before endocardial leads are placed or alternatively an epicardial device should be implanted. It remains unclear how to exactly manage patients with a small shunt at an atrial level across a patent foramen ovale and further studies would be needed to develop recommendations [21]. It is also vital to recognize that the patients with a classic Fontan operation frequently have very large atria and slow flow through their neo-chamber. Thus, it is not uncommon for large thrombi with a potential for pulmonary thromboembolism to develop on transvenous leads. And although this does not present an absolute contraindication [69, 87], it does need to be carefully considered and weighed against the risks of epicardial lead placement. Long-term oral anticoagulation does also need to be accordingly considered in this group.

#### Lead position

The pacing site of the ventricular leads is a critical issue, as it has a major impact on left ventricular mechanical synchrony, efficiency, and pump function in children who require lifelong pacing. The right ventricular apex has been the most used pacing site, because it is easily accessible transvenously and provides a stable lead position with a low dislodgment rate. However, long-term right ventricular apical pacing induces an iatrogenic left bundle branch block and can lead to pacing-induced cardiomyopathy with left ventricular dilation and both systolic and diastolic left ventricular dysfunction, for both endocardial and epicardial leads [46, 67, 86, 91, 99]. There is early encouraging data that suggest improved overall hemodynamics and ventricular function by cardiac resynchronization therapy with single-site left ventricular pacing via the coronary sinus in this group [41]. Until further supportive evidence is accrued, it is important for the implanter and clinician to consider this approach in any patient whose ejection fraction is suboptimal.

However, detrimental effects of the right ventricular apical pacing led to the reassessment of traditional approaches and to the research of alternative pacing sites, in order to get to more physiological pattern of ventricular activation. Although being theoretically the best technique, direct His-Bundle pacing and paraHisian cardiac pacing remain complex and associated with higher pacing thresholds are required, causing accelerated battery depletion [93]. Right ventricular septal pacing is a good alternative, technically easy and maintaining a lower pacing threshold [22]. By preserving septal to lateral left ventricular synchrony and systolic function, left ventricular apical and midlateral wall pacing may be the preferred pacing site for epicardial leads in the young [41, 81].

#### Mode of pacing

A five-letter international code describes pacemaker function, three letters being in common usage: the first letter is the chamber paced (A = atrium; V = ventricle; D = dual, namely both A and V; O = none), the second letter is the chamber sensed and the third letter describes the algorithm used to integrate pacing and sensing functions. Most of children and teenagers will benefit from a dual-chamber pacing (DDD), allowing AV synchrony that is important to maintain ventricular filling and stroke volume [89, 96]. In infants with complete AV block and normal sinus node function, a single-chamber ventricular pacing (VVI or VDD) should be selected, because it requires only a unique lead (uni- or bipolar) thus reducing the risk of venous occlusion [89, 96].

In patients with conduction system disease undergoing pacemaker implantation, coexistent reentrant atrial tachycardias are common. Therefore, the implantation of devices with atrial antitachycardia pacing capability is reasonable [84]. This approach is more common as an adjunct to maze procedures and in conjunction with antiarrhythmic therapy [92].

#### Perspectives

To overcome the potential short- and long-term complications related to transvenous leads and subcutaneous pulse generators, new technologies enabling leadless cardiac stimulation have been developed and proved to be applicable and safe in experimental models and in some small human studies [52, 71, 82]. These ultrasound or induction technologies, based on leadless pacemakers, could be used to provide cardiac stimulation from the endocardium at selected sites. The absence of a transvenous lead and subcutaneous pulse generator could represent a paradigm shift in cardiac pacing. Future studies will need to address the safety/efficacy of alternate-site RV and LV pacing and the long-term outcomes of these new approaches. Although the continued development and success of electronic pacing are impressive, gene therapy for biological pacing is also a promising research field. This approach might offer an alternative treatment for pacemaker-related infections in the future, with right ventricular intramyocardial injection of an adenoviral construct whose duration of effect is temporary, until such time as the infection is adequately treated and a new permanent pacemaker can be inserted [35, 74].

## Conclusion

Although rare, congenital and childhood AV block is important treatable cause of morbidity and mortality in the young. This review crystallizes our contemporary understanding and management strategies, highlighting the ongoing investigation in all aspects of care related to this complex group of disorders. Much work is needed, especially with regards to the antenatal detection and treatment of congenital AV block, and on a genetic level, and it is evident that as new insights are gleaned, management strategies continue to evolve. Despite the many different etiologies, each with specific management and distinct outcomes, these patients can do well with a careful, considered approach to the diagnostic evaluation and management plan.

## Abbreviations

ACC: American College of Cardiology
AHA: American Heart Association
AV: Atrioventricular
CHB: Congenital heart block
CHD: Congenital heart disease
DCM: Dilated cardiomyopathy
ESC: European Society of Cardiology
HRS: Heart Rhythm Society
PCCD: Progressive cardiac conduction disease
SVC: Superior vena cava
